# A realworld pharmacovigilance study of trazodone based on the FDA adverse event reporting system

**DOI:** 10.1038/s41598-025-89632-7

**Published:** 2025-02-13

**Authors:** Yong Yu, Xin Sun, Liqun Hao, Xiaoyan Zhang, Yankui Guo

**Affiliations:** 1 The First Clinical College of Medicine, Shandong University of Traditional Chinese Medicine, Jinan, 250013 Shandong Province China; 2 Jinan Hospital of Guang an men Hospital, China Academy of Chinese Medical Sciences, Jinan, 250012 Shandong Province China

**Keywords:** Trazodone, FDA adverse event reporting system, Adverse drug reaction, Data mining, Drug safety, Circadian rhythms and sleep, Emotion

## Abstract

To explore and analyze the potential adverse event (AE) signals of trazodone, with reference to the safe clinical use of drugs. Based on the FDA Adverse Event Reporting System (FAERS), the AE data of trazodone were extracted from the first quarter of 2004 to the second quarter of 2024, and the extracted data were statistically analyzed using the method, to identify valid AE signals that met our judgment, compare them with those recorded in the authorized information for trazodone thereby identifying unexpected potential adverse reactions. A total of 5199 AE reports with trazodone as the main suspect were extracted, with a higher reported proportion of females (52.68%) than males (38.83%). Many reports (31.47%) did not provide age information, although for those reports with identifiable age data, the 50–60 years age group was the most common (14.20%), and the country of reporting was predominantly the United States (82.58%). A total of 179 significant AE signals were unearthed, with suicide, formulation toxicity, abnormal penile erection, insomnia, and cardiac and respiratory arrest reported with high frequency, which was not entirely consistent with the specification record. The study unearthed 156 new potential AEs on the basis of trazodone drug inserts and suggested precautions for overdosing and dose adjustment, which is conducive to safeguarding the safety of patients’ medication.

**Supplementary Information:**

The online version contains supplementary material available at 10.1038/s41598-025-89632-7.

## Introduction

Developed as a second-generation antidepressant in the 1960s by Angelini Research Laboratories in Italy, trazodone was the first non-tricyclic antidepressant approved for use in the United States in 1981^1^. As a triazolopyridine derivative, trazodone belongs to the group of 5-hydroxytryptamine (5-HT_2_) antagonists/reuptake inhibitors (SARIs)^2^, acting on a variety of neurotransmitter receptors and blocking the 5-HT transporter (serotonin transporter, SERT), 5-HT_2_ receptor, adrenergic α1 receptor, and histamine receptor 1 (H_1_)^3,4^.

Trazodone is traditionally considered an “old-fashioned” antidepressant; however, in a multidisciplinary context, new therapeutic advantages are being explored due to its specific pharmacological profile. Trazodone has a faster onset of action than most antidepressants with an onset of action of less than one week, filling the gap between the onset of action of selective serotonin reuptake inhibitors (SSRIs) and serotonin/norepinephrine reuptake inhibitors (SNRIs)^5,6^. Furthermore, it is pharmacodynamically malleable, preferentially antagonizing different receptors at various doses to target varying depressive symptoms, including depression, anxiety, and insomnia^2^. It is also pharmacokinetically tolerable, exhibiting fewer interactions when used in combination with other medications to treat co-morbid depressive symptoms, thereby achieving complementary effects^7^. Despite the general consensus that trazodone is a relatively safe and effective antidepressant, events, including common ones such as sedation, dizziness, headache, and postural hypotension, have been reported^8^. Additionally, specific adverse events such as Parkinson’s disease, injurious fall risk, and treatment-resistant priapism have attracted attention^5,7,9^. These side effects can affect adherence to treatment and may lead to self-discontinuation of the drug by the patient, heightening the risk of adverse outcomes. Moreover, the short observation period of many clinical studies and the lack of comprehensive long-term risk monitoring of trazodone may underestimate its side effects.

The clinical use of trazodone requires a multidisciplinary approach, involving psychiatry, neurology, and geriatrics, as well as ensuring the safety of co-prescribing. A comprehensive understanding of its potential adverse effects and the assessment of the risks and benefits of treatment are essential to facilitate the individualized treatment of depression. Traditional single-study approaches are often ineffective in capturing the intricate associations between drugs and adverse reactions, comprehensive knowledge of their adverse effects is still lacking. The FDA Adverse Event Reporting System (FAERS) is a comprehensive database containing information on adverse events (AEs) and drug error reports submitted to the FDA. It serves as a valuable resource for post-marketing safety surveillance, enabling healthcare professionals and the public to report adverse reactions associated with drug use^10,11^. Therefore, the aim of this study was to assess the relationship between trazodone and reported adverse reactions from multiple perspectives by utilizing the extensive dataset from the FAERS database. The safety of trazodone was thoroughly evaluated to furnish valuable insights for clinical decision-making.

## Methods

### Data sources

Reports of AEs from the first quarter of 2004 to the second quarter of 2024 were collected. For data entries with identical case IDs in the demographic and administrative information (DEMO) table, only the most recent report was retained, as determined by the report date. Trazodone’s involvement in AEs was meticulously categorized as a primary suspect (PS), secondary suspect, concomitant, or interacting agent. Special attention was given to reports that designated trazodone as the primary suspect, indicating its potential contribution to AEs. These reports included clinical characteristics such as patient age and gender, the identity of the reporter, the country of origin, and the outcome. For the initial screening of this study, Preferred Terms (PTs) with a reported count of three or more were selected. The AE signals were coded using PTs from the Medical Dictionary for Regulatory Activities (MedDRA) version 26.1 and categorized by System Organ Class (SOC) to analyze the specific SOC involved in the AE signal. The data were imported into the R software (version 4.4.1) for processing.

### Data extraction and analysis

Quantitative data mining methods for spontaneous AE reports are generally based on the calculation of disproportionality measures. This method is a key technique in pharmacovigilance studies, employing 2 × 2 contingency tables (Supplementary Table S1), and plays a crucial role in identifying potential signals of drug-related AEs. The rationale behind these methods is to compare the frequency of occurrence of the target event with its frequency when occurring with all other drugs; a significant signal is generated when both the frequency and signal intensity of the target drug and the AE exceed a specified threshold^12^.

**Fig. 1:**
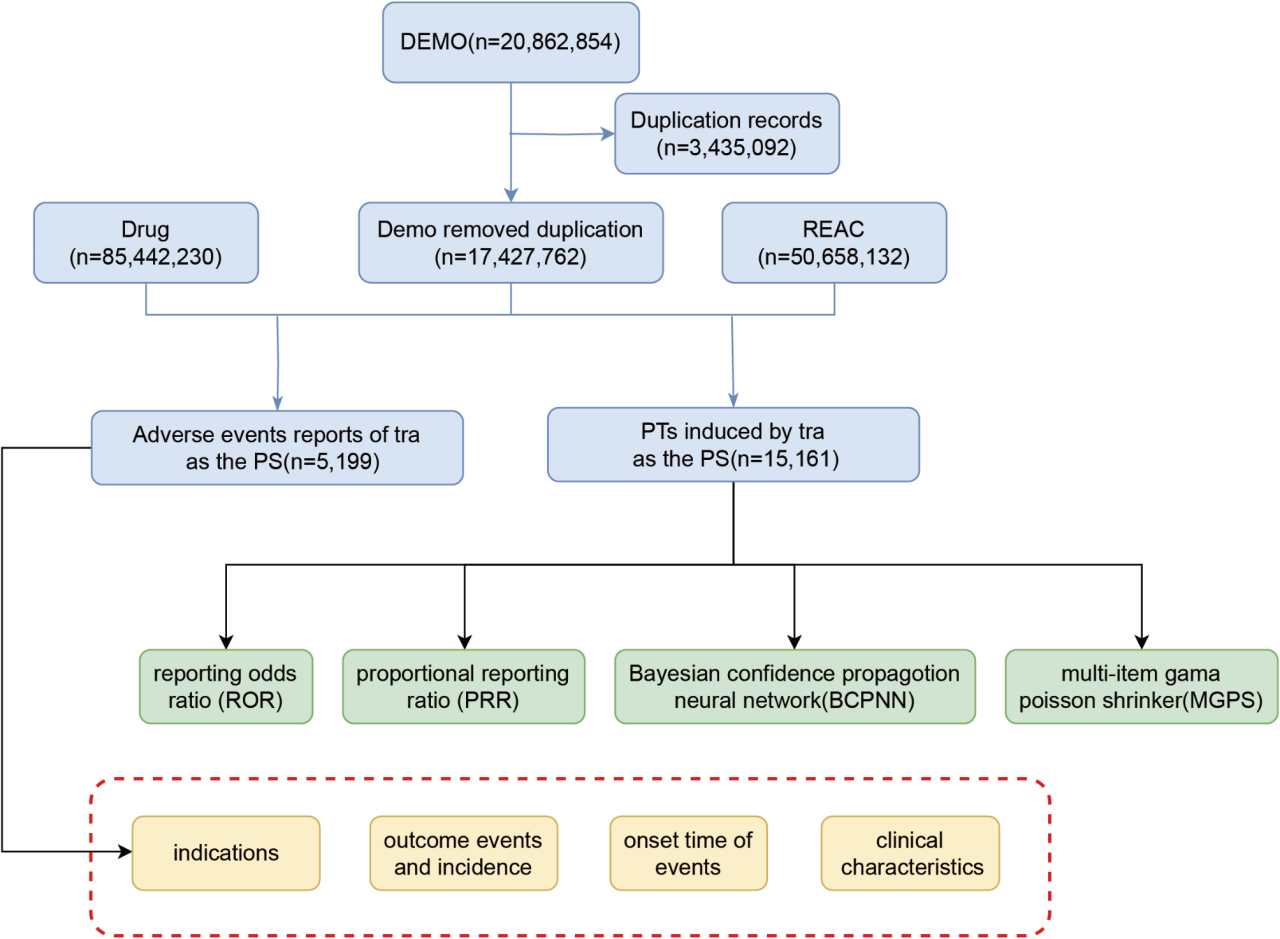
The flow diagram of selecting trazodone-related AEs from FAERS database.

First, the common association metrics, Reporting Odds Ratio (ROR) and Proportional Reporting Ratio (PRR), were used. These methods have the advantage of high sensitivity^13^; however, they are unstable when there are few reports of specific drug-event combinations^14^. To overcome this shortcoming, the study used the Multi-Item Gamma Poisson Shrinker (MGPS) approach, which employs empirical Bayesian modeling to systematically identify and “shrink” very common and unstable observations, facilitating the detection of signals from rare event^15^. The MGPS can also adjust for random noise using a model derived from the data and correct for temporal tendencies and confounders associated with variables such as age and gender by stratifying over 900 categories. In addition, we employed the Bayesian Confidence Propagation Neural Network (BCPNN), which utilizes Bayesian statistics and neural network methodologies to enhance the early detection of AE signals^16^. The combined use of these four algorithms is intended to minimize bias inherent in single-method analyses and to detect safety signals more comprehensively and reliably. Specific formulas and thresholds are detailed in Supplementary Table S2. Signals of AEs can be identified when the results of all four analytical methods yield significance. Higher signal intensity values indicate a closer association between the target drug and the AE. Figure 1 illustrates the data mining process.

## Results

### Basic characteristics of trazodone-related AEs

From the first quarter of 2004 to the second quarter of 2024, a total of 17,427,762 AE reports from the FAERS database were acquired for this study. Of these, 5199 reports identified trazodone as the main suspected drug for AEs, involving 15,161 AEs. Table 1 demonstrates the clinical characterization information for AEs reports of trazodone. AE reports with collected demographic data showed a significant predominance of reported female patients over male patients (52.68% vs. 38.83%). A significant proportion of the data (31.47%) lacked age information, and among the reports with explicit age data, AEs were most frequent in patients aged 50–60 years (14.20%). Notably, consumers rather than healthcare professionals submitted the majority of the reports (36.99%). The U.S. accounted for the highest proportion of reports (82.58%). The most common indication for trazodone treatment was insomnia (18.04%), although a subset of patients also received it for depression (9.17%). Regarding clinical outcomes, apart from unspecified serious AEs, hospitalization was the most frequently reported AE (26.23%), followed by death (22.85%). Of the AEs with a known time of occurrence, a significant number occurred within 7 days of dosing (30.12%).

**Table 1:** Basic information on adverse reaction reports related to trazodone.

Clinical characteristics	Number of reports	Percentage (%)
Year		
2004	138	2.65
2005	115	2.21
2006	122	2.35
2007	87	1.67
2008	105	2.02
2009	188	3.62
2010	132	2.54
2011	193	3.71
2012	95	1.83
2013	114	2.19
2014	203	3.9
2015	376	7.23
2016	300	5.77
2017	309	5.94
2018	460	8.85
2019	357	6.87
2020	489	9.41
2021	434	8.35
2022	328	6.31
2023	391	7.52
2024	263	5.06
Gender		
Female	2739	52.68
Male	2019	38.83
Unknown	441	8.48
Age		
< 30	475	9.14
30 ~ 40	440	8.46
40 ~ 50	672	12.93
50 ~ 60	738	14.2
60 ~ 70	610	11.73
70 ~ 80	373	7.17
>=80	255	4.9
Unknow	1636	31.47
Reporter		
Consumer	1923	36.99
Physician	1193	22.95
Pharmacist	1112	21.39
Other health-professional	623	11.98
Unknown	335	6.44
Lawyer	12	0.23
Registered Nurse	1	0.02
Reported countries (Top 5)		
United States	3341	82.58
United Kingdom	200	4.94
Canada	183	4.52
Other	78	1.93
Spain	55	1.36
Route		
Other	3005	57.8
Oral	2166	41.66
Transplacental	28	0.54
Outcomes		
Other serious	1927	38.94
Hospitalization	1298	26.23
Death	1131	22.85
Life threatening	226	4.57
Disability	195	3.94
Required intervention to Prevent Permanent Impairment/Damage	158	3.19
Congenital anomaly	14	0.28
Time to event onset (days)		
< 7	534	30.12
7 ~ 30	181	10.21
30 ~ 180	166	9.36
180 ~ 360	52	2.93
>=360	150	8.46
Unknow	690	38.92
Indications		
Affective disorder	13	0.24
Agitation	15	0.28
Antidepressant therapy	36	0.68
Anxiety	101	1.89
Anxiety disorder	11	0.21
Bipolar disorder	22	0.41
Depression	489	9.17
Drug abuse	64	1.2
Insomnia	962	18.04
Major depression	57	1.07
Others	293	5.49
Pain	17	0.32
Post-traumatic stress disorder	30	0.56
Product used for unknown indication	1348	25.28
Schizophrenia	14	0.26
Sleep disorder	473	8.87
Sleep disorder therapy	32	0.6
Somnolence	24	0.45
Suicide attempt	132	2.48
Unknown	1200	22.5

### Trazodone signal mining

In our analysis of the FAERS database, 24 SOCs were identified as involved in the AEs associated with trazodone. The three most frequently reported AE categories included psychiatric disorders (*n* = 3023, ROR 3.91, PRR 3.33, IC 1.74, EBGM 3.33), general disorders and administration site conditions (*n* = 2244, ROR 0.78, PRR 0.81, IC -0.3, EBGM 0.81), nervous system disorders (*n* = 2148, ROR 1.69, PRR 1.6, IC 0.67, EBGM 1.59) (Table 2). The categories with the strongest signals are reproductive system and breast disorders (*n* = 507, ROR 3.97, PRR 3.87, IC 1.95, EBGM 3.86). These findings correlate with the SOCs listed for common AEs in the drug inserts, indicating a high level of data reliability.

**Table 2:** Signal intensity of trazodone AEs at SOC level.

SOC	Case reports	ROR (95% CI)	PRR (95% CI)	chisq	IC (IC025)	EBGM (EBGM05)
Psychiatric disorders	3023	3.91 (3.76, 4.07)	3.33 (3.2, 3.46)	5246.75	1.74 (1.68)	3.33 (3.22)
General disorders and administration site conditions	2244	0.78 (0.75, 0.82)	0.81 (0.78, 0.84)	115.28	− 0.3 (− 0.36)	0.81 (0.78)
Nervous system disorders	2148	1.69 (1.62, 1.77)	1.6 (1.54, 1.66)	523.37	0.67 (0.61)	1.59 (1.54)
Injury, poisoning and procedural complications	1663	1.16 (1.1, 1.22)	1.14 (1.1, 1.19)	31.99	0.19 (0.12)	1.14 (1.09)
Gastrointestinal disorders	982	0.71 (0.66, 0.75)	0.73 (0.69, 0.77)	111.99	− 0.46 (− 0.56)	0.73 (0.69)
Investigations	705	0.7 (0.65, 0.76)	0.72 (0.67, 0.78)	84.24	− 0.48 (− 0.59)	0.72 (0.67)
Respiratory, thoracic and mediastinal disorders	692	0.91 (0.85, 0.98)	0.92 (0.85, 1)	5.59	− 0.13 (− 0.24)	0.92 (0.86)
Cardiac disorders	674	1.62 (1.5, 1.75)	1.59 (1.47, 1.72)	150.94	0.67 (0.56)	1.59 (1.49)
Reproductive system and breast disorders	507	3.97 (3.63, 4.33)	3.87 (3.58, 4.19)	1086.34	1.95 (1.82)	3.86 (3.59)
Skin and subcutaneous tissue disorders	450	0.52 (0.47, 0.57)	0.53 (0.48, 0.58)	198.49	− 0.92 (− 1.05)	0.53 (0.49)
Musculoskeletal and connective tissue disorders	443	0.51 (0.47, 0.57)	0.53 (0.48, 0.58)	196.71	− 0.92 (− 1.05)	0.53 (0.49)
Eye disorders	303	0.96 (0.86, 1.07)	0.96 (0.85, 1.08)	0.54	− 0.06 (− 0.22)	0.96 (0.87)
Metabolism and nutrition disorders	253	0.74 (0.65, 0.84)	0.74 (0.66, 0.83)	22.86	− 0.43 (− 0.61)	0.74 (0.67)
Vascular disorders	229	0.66 (0.58, 0.75)	0.67 (0.58, 0.77)	38.88	− 0.58 (− 0.77)	0.67 (0.6)
Infections and infestations	185	0.21 (0.18, 0.25)	0.22 (0.19, 0.25)	532.15	− 2.17 (− 2.38)	0.22 (0.2)
Renal and urinary disorders	171	0.58 (0.5, 0.67)	0.58 (0.5, 0.68)	51.44	− 0.77 (− 0.99)	0.58 (0.52)
Hepatobiliary disorders	121	0.83 (0.7, 1)	0.84 (0.7, 1)	3.93	− 0.26 (− 0.52)	0.84 (0.72)
Immune system disorders	117	0.66 (0.55, 0.8)	0.67 (0.56, 0.8)	19.62	− 0.58 (− 0.84)	0.67 (0.57)
Blood and lymphatic system disorders	83	0.3 (0.25, 0.38)	0.31 (0.25, 0.38)	130.99	− 1.7 (− 2.01)	0.31 (0.26)
Ear and labyrinth disorders	73	1.07 (0.85, 1.34)	1.07 (0.85, 1.35)	0.32	0.09 (− 0.23)	1.07 (0.88)
Pregnancy, puerperium and perinatal conditions	33	0.48 (0.34, 0.68)	0.48 (0.34, 0.67)	18.53	− 1.05 (− 1.54)	0.48 (0.36)
Endocrine disorders	28	0.7 (0.48, 1.01)	0.7 (0.48, 1.02)	3.64	− 0.52 (− 1.04)	0.7 (0.51)
Congenital, familial and genetic disorders	25	0.51 (0.34, 0.75)	0.51 (0.34, 0.75)	11.82	− 0.97 (− 1.53)	0.51 (0.37)
Neoplasms benign, malignant and unspecified (incl cysts and polyps)	9	0.02 (0.01, 0.04)	0.02 (0.01, 0.04)	416.95	− 5.55 (− 6.45)	0.02 (0.01)

**Fig. 2:**
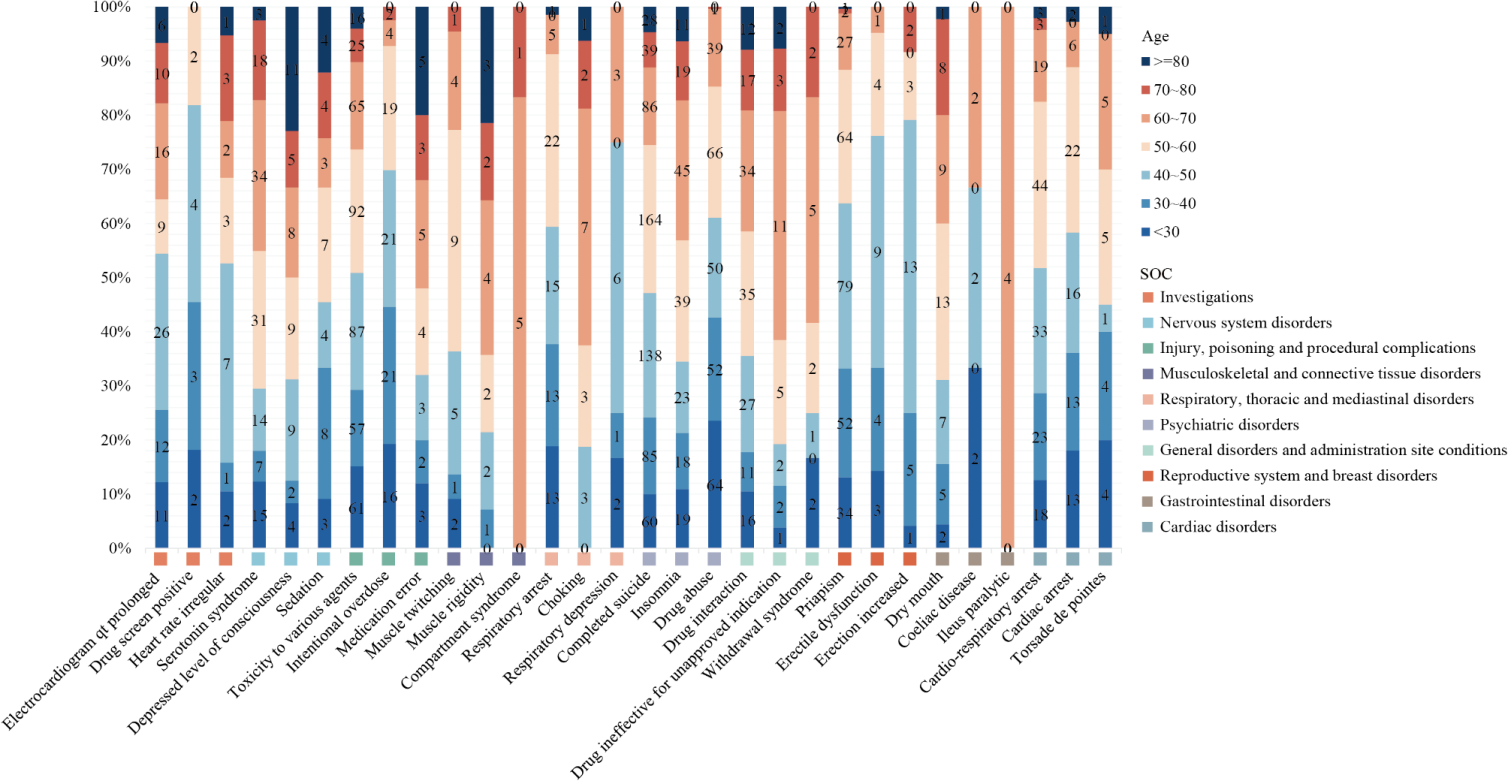
Age distribution of common adverse events across system organ classes.

At the PT level, four algorithms were employed to analyze trazodone-related AEs and evaluate their compliance with the screening criteria, identifying a total of 217 PTs. The top 20 PTs were ranked according to the number of reports, as detailed in Table 3. The results revealed the most commonly reported PTs to include completed suicide (*n* = 610, ROR 27.9, PRR 26.82, IC 4.73, EBGM 26.61), toxicity to various agents (*n* = 412, ROR 9.96, PRR 9.72, IC 3.28, EBGM 9.69) and priapism (*n* = 332, ROR 404.77, PRR 395.93, IC 3.28, EBGM 354.08). Notably, some adverse reactions are not mentioned in the drug inserts, including completed suicide, formulation toxicity, and cardiac and respiratory arrests, which occurred with a higher frequency and signal intensity, which may require further attention and research. Additionally, we analyzed the age distribution characteristics of common PTs in various SOCs (Fig. 2). AEs such as priapism and erectile dysfunction were prevalent in the 40–50 years age group, whereas completed suicide and cardio-respiratory arrest occurred most frequently in the 50–60 years age group.

**Table 3:** Top 20 PTs ranked by number of trazodone AEs.

SOC	PT	Case reports	ROR (95% CI)	PRR (95% CI)	chisq	IC (IC025)	EBGM (EBGM05)
Psychiatric disorders	Completed suicide	610	27.9 (25.72, 30.26)	26.82 (24.8, 29.01)	15063.79	4.73 (4.62)	26.61 (24.86)
Injury, poisoning and procedural complications	Toxicity to various agents	412	9.96 (9.03, 10.99)	9.72 (8.81, 10.72)	3221.5	3.28 (3.14)	9.69 (8.93)
Reproductive system and breast disorders	Priapism	332	404.77 (360.83, 454.05)	395.93 (352, 445.34)	116933.08	8.47 (8.3)	354.08 (321.62)
Psychiatric disorders	Insomnia	293	4.19 (3.73, 4.7)	4.13 (3.67, 4.65)	696.76	2.04 (1.88)	4.12 (3.74)
Psychiatric disorders	Drug abuse	275	12.66 (11.24, 14.27)	12.45 (11.07, 14)	2889.2	3.63 (3.46)	12.41 (11.23)
General disorders and administration site conditions	Drug interaction	169	4.04 (3.48, 4.71)	4.01 (3.43, 4.69)	382.57	2 (1.78)	4.01 (3.53)
Cardiac disorders	Cardio-respiratory arrest	146	12.77 (10.84, 15.03)	12.65 (10.81, 14.8)	1561.99	3.66 (3.42)	12.6 1(11)
Psychiatric disorders	Confusional state	145	3.4 (2.89, 4)	3.38 (2.89, 3.95)	242.8	1.75 (1.52)	3.37 (2.94)
Nervous system disorders	Serotonin syndrome	142	29.63 (25.1, 34.98)	29.36 (25.1, 34.34)	3857.72	4.86 (4.63)	29.12 (25.34)
Investigations	Electrocardiogram qt prolonged	102	11.07 (9.11, 13.45)	11 (9.04, 13.38)	924.76	3.46 (3.18)	10.97 (9.32)
Psychiatric disorders	Suicide attempt	94	5.88 (4.8, 7.21)	5.85 (4.81, 7.12)	378.09	2.55 (2.26)	5.85 (4.93)
Injury, poisoning and procedural complications	Intentional overdose	87	5.46 (4.42, 6.74)	5.43 (4.38, 6.74)	314.58	2.44 (2.14)	5.43 (4.55)
Psychiatric disorders	Agitation	80	4.03 (3.24, 5.03)	4.02 (3.24, 4.99)	181.42	2.01 (1.69)	4.02 (3.34)
Cardiac disorders	Cardiac arrest	76	3.43 (2.74, 4.29)	3.41 (2.75, 4.23)	129.83	1.77 (1.45)	3.41 (2.83)
General disorders and administration site conditions	Drug ineffective for unapproved indication	74	5.49 (4.37, 6.9)	5.47 (4.32, 6.92)	269.9	2.45 (2.12)	5.46 (4.51)
Psychiatric disorders	Nightmare	71	7.68 (6.08, 9.7)	7.65 (6.05, 9.68)	409.54	2.93 (2.6)	7.63 (6.28)
Gastrointestinal disorders	Dry mouth	71	3.42 (2.7, 4.31)	3.4 (2.69, 4.3)	120.6	1.77 (1.43)	3.4 (2.8)
Respiratory, thoracic and mediastinal disorders	Respiratory arrest	70	9.03 (7.13, 11.42)	8.99 (7.11, 11.37)	495.92	3.16 (2.83)	8.97 (7.37)
Respiratory, thoracic and mediastinal disorders	Choking	53	10.64 (8.12, 13.94)	10.6 (8.06, 13.95)	459.75	3.4 (3.02)	10.57 (8.44)
Injury, poisoning and procedural complications	Medication error	50	3.46 (2.62, 4.56)	3.45 (2.62, 4.54)	86.94	1.79 (1.39)	3.45 (2.73)

## Discussion

### Main findings of the study

Analysis of gender data in trazodone case reports showed that females accounted for a greater proportion of AE reports, which may be associated with a higher risk of depression in females than in males, leading to greater use of trazodone in females^17^. Analysis of age-related data showed that the 50–60 age group accounted for a larger proportion of AE reports suggesting that the drug may be more widely used in the 50–60 age group or that this age group may be predisposed to the drug. The highest percentage of reports from the United States was 82.58%, followed by the United Kingdom, Canada, and Japan, which were mainly concentrated in Europe and the United States, this is likely because the FAERS database is a self-reporting system, and the reporting regions are predominantly the U.S. and Europe. Additionally, the limited reports of adverse events from Asia may have introduced selection bias. Among the reported outcomes of adverse events, the most serious was hospitalization, although there were other reported serious outcomes. In terms of the time of occurrence of adverse events, the highest frequency of adverse events occurred within 7 days of dosing, where that information had been provided. In addition to known adverse events, new unexpected events such as completed suicide, cardiac and respiratory arrests, and fluctuating states of consciousness were identified. These findings may provide valuable information for future drug safety assessments and further studies are needed to confirm the above findings given the well-known limitations of spontaneous reports.

### Comparison with the drug label

This study identified a series of risk signals for AEs, and based on disproportionality analysis, the AEs for trazodone predominantly involved psychiatric disorders, which is consistent with its drug insert and clinical study reports. Analyzing the adverse events reported for the drug, the adverse events for trazodone were predominantly included in the drug label. This suggests that common adverse events occurring after the drug is marketed are consistent with the drug labeling, with obvious exceptions such as suicide, substance abuse, and cardiac arrest. In addition, some of the adverse events in the top 20 signal strengths are not mentioned in drug labels after eliminating product use problems not associated with to the drug itself, various surgical and medical manipulations, and socio-environmental signals, suggesting the presence of a number of new suspected high-risk signals. These should be emphasized in the clinical setting.

### New AE signals

Suicide: it has been suggested that trazodone is an effective option to reduce the risk of suicide in major depressive disorder due to its efficacy as an antidepressant in reducing depressive co-morbidities such as anxiety, mania, and insomnia^18^. In our results, completed suicide emerged as the AE signal with the highest incidence, which does not entirely align with existing studies and should be a cause for significant concern.

Suicidal ideation was significantly associated with the severity of depression in half of the clinically depressed patients^19^. In a meta-analysis, it was noted that the prevalence of suicidal ideation and suicide attempts in patients with major depression was 53.1% and 31%, respectively^20,21^. Trazodone is commonly used in the treatment of patients with major depression, and such patients are inherently at high risk of suicide. Trazodone, as an antidepressant, even at standard therapeutic doses, can lead to overstimulation of the central nervous system, which can have deleterious effects, including the risk of suicide. A major constraint of antidepressant therapy is the slow onset of action; typically, the initial effects of most antidepressant medications begin to be observed within 2 weeks, and full effect may not be achieved until 6 to 8 weeks^22^. Patients with depression have an increased risk of suicide during the first month, or even the first week, of treatment^23,24^, and delayed treatment effects may be an important reason for the increased risk of suicide. Clinical studies have shown that trazodone produces antidepressant effects faster compared to venlafaxine, bupropion, and fluoxetine^6,25,26^, and is an effective option for reducing the risk of suicide in major depression. However, mitigating the risk of suicide in patients with major depression is not an easy task, and our findings emphasizes the importance of clinicians monitoring patients for suicidal ideation while they are taking trazodone.

Priapism: In this study, priapism is the most common adverse event of reproductive system. Priapism is characterized by a persistent, often painful penile erection unrelated to sexual stimulation lasting more than four hours^27^. Drug-induced priapism is the most common cause of this condition, with approximately 50% of drug-related cases attributed to antipsychotic use^28^. The mechanism by which trazodone induces priapism is not fully understood, with the most common hypothesis involving α-adrenergic blockade of the corpora cavernosa of the penis^29^. Priapism represents a significant urologic emergency; if left untreated, it may lead to hypoxia-related destruction of the sinusoidal endothelium and corporal fibrosis, ultimately resulting in permanent erectile dysfunction^27^, and in patients with a history of comorbid venous thrombosis, even the risk of penile amputation^30^. Priapism may manifest during long-term treatment as well as at the first administration, involving different drugs and low starting doses^31^. Patients at high risk of developing this condition include those with sickle cell anemia, leukemia, autonomic nervous system dysfunction, hypercoagulability, and those taking SSRIs^32^. Our findings provide important insights into trazodone’s safety profile, suggesting that alternative therapies should be considered for patients who experience priapism or have associated risk factors. Physicians should inform patients about the possibility of priapism and its sequelae and encourage early intervention to avoid irreversible consequences.

Cardiovascular Disease: There is an established bidirectional link between depression and cardiovascular disease. Traditionally, trazodone has been recommended for patients with cardiovascular disease due to its reputed cardiovascular safety profile in clinical practice^33^. However, this safety has been questioned as several studies have raised concerns regarding trazodone’s cardiotoxic potential^34–36^. In our study, trazodone was also found to pose a risk of causing prolongation of the QT interval, torsade de pointe, and cardiac arrest. The precise mechanism remains unclear; however, the prevailing explanation is that trazodone overdose inhibits cardiac HERG potassium channels, leading to prolonged repolarization of cardiomyocytes, which increases the risk of cardiac arrhythmias^37^. This suggests that clinicians should be cautious when prescribing trazodone to patients with cardiac disease and should advise them to monitor the electrocardiogram, heart rate, and other indicators while taking the drug.

Other adverse events. Psychiatric: drug abuse, intentional overdose. This may be associated with suicidal ideation in depressed patients. Confusional state and delirium: patients and their caregivers should be instructed to observe any alterations in consciousness and report them to healthcare providers promptly. Dyskinesia: case reports have documented movement disorders such as orofacial dyskinesia and restless legs syndrome induced by trazodone use^38,39^. The mechanism may involve the antagonistic effect of trazodone on dopamine receptors^40^. Respiratory arrest and choking: the mechanism of which is currently unknown.

### Clinical significance and limitations

The results of this study are an important warning for clinicians, emphasizing the need for caution and more rigorous patient monitoring during use. However, the study has some limitations. First, the FAERS database, as an autonomous reporting system, may be constrained by external factors such as reduced reporting and duplicate reporting, which do not reflect the full range of adverse reactions in the real world, making it more difficult to accurately assess drug safety. Second, AE signals detected using the disproportionality methods only demonstrate a statistical correlation with the target AE, not biological causation. Therefore, further clinical investigation is necessary to determine causation. Reports often lack adequate detail, such as statistical information on different doses and dosage forms, for a more in-depth evaluation. An overemphasis on product risks can lead to an exaggerated perception of drug-related risks, especially when the total number of patients using the product is unknown.

## Conclusion

This study’s comprehensive pharmacovigilance analysis of the FAERS database adds evidence based on real-world data for the clinical safety management of trazodone. This study confirmed several known AEs, such as insomnia, nightmare, and dry mouth, and identified several new potential AEs, including suicide, drug abuse, priapism, and cardiac and respiratory arrest, etc. The multilevel analysis of this study provides a vigilant reference for clinicians to optimize drug selection and safety monitoring. More rigorous prospective, multicenter clinical trials and epidemiological studies are still needed in the future to validate our findings for a more precise assessment of the safety risks of trazodone.

## Electronic supplementary material

Below is the link to the electronic supplementary material.

Supplementary Material 1

## Data Availability

The datasets generated during and analyzed during the current study are available from the corresponding author on reasonable request.
